# Draft genome sequence of probiotic bacterium *Lactobacillus fermentum* S2, isolated from raw cow milk

**DOI:** 10.1128/MRA.00499-23

**Published:** 2023-10-31

**Authors:** Rajarshi Bhattacharya, Bidhan Chandra Mukhopadhyay, Mirambika Mishra, Ananta Prasad Arukha, Sukhendu Mandal, Swadesh Ranjan Biswas

**Affiliations:** 1 Molecular Food Microbiology Laboratory, Department of Botany, Siksha Bhavana, Visva-Bharati, Santiniketan, India; 2 Department of Infectious Disease and Immunology, College of Veterinary Medicine, University of Florida, Gainesville, Florida, USA; 3 Department of Nephrology and Hypertension, Mayo Medical Science Building, Rochester, Minnesota, USA; 4 Laboratory of Molecular Bacteriology, Department of Microbiology, University of Calcutta, Kolkata, India; Indiana University, Bloomington, Indiana, USA

**Keywords:** probiotics, lactic acid bacteria, fermentation, milk

## Abstract

*Lactobacillus fermentum* remains as potential probiotic bacterium that enhances immunological response, produces antimicrobials, acts as food preservative, and lowers blood cholesterol level. We report the draft genome of *Lactobacillus fermentum* S2 consisting of 1.97 Mb genome size, 52.27% G + C content, 3 rRNA genes, 51 tRNA genes, and 2,004 protein-coding sequences.

## ANNOUNCEMENT

The gut and fermented foods are the natural habitats for the probiotic bacteria that play an important role in our lives and even extend our lifespan (1). Current research documents the alteration of probiotic gut bacteria in almost all human diseases, including COVID-19 (2). This change can affect both the gut and other human organs. Therefore, maintaining a balance of probiotic bacteria is essential for a healthy life. Lactic acid bacteria (LAB), under the phylum Firmicutes, are also used in the production of various fermented foods such as cottage cheese, kefir, butter, buttermilk, curd, koumiss, sausage, sauerkraut, and yogurt. Using specific probiotic starter cultures when making these fermented foods could be a promising way to get them into the gut and prevent dysbiosis (3).

Probiotic application in food and health is purely strain-specific, and decoding information through genome sequencing is important to harness it for human benefit. Among the LAB, *Lactobacillus* remains the best-studied genus. *L. fermentum* is one of the species of the genus *Lactobacillus*, that is widespread among fermented foods. In view of its immense potential, a strain of *L. fermentum* S2 was isolated from untreated Indian breed cow milk in Santiniketan, West Bengal, India (23.6776^0^N, 87.6853^0^E), and the genome of this strain was sequenced to study for milk fermentation and the probiotic potential. The strain S2 was obtained by culturing on de Man, Rogosa, and Sharpe (MRS) medium after the standard dilution method (4). The resulting single colony was sub-cultured by repeated streaking onto MRS agar plates followed by incubation for 48 h at 30°C (5). Genomic DNA was isolated, and the 16S rRNA was amplified using the 8F and 1,492R universal primers, following established procedure (6). The PCR amplicons were purified and sequenced, followed by sequence analysis utilizing the BLASTn tool (https://blast.ncbi.nlm.nih.gov). Phylogenetic tree was constructed by neighbour joining (NJ) using Mega 11 (7). The 16S rRNA gene sequence similarities between strains were calculated on the basis of pair-wise alignment using the NCBI BLASTn (https://blast.ncbi.nlm.nih.gov/Blast.cgi). Strain S2 showed maximum similarity (100%) with *L. fermentum* Strain SK152. For whole-genome sequence of the strain, the genomic DNA of the pure culture of S2 was extracted using a Qiagen genomic-tip 100/G following the manufacturer’s instruction (8), and the genomic DNA library was prepared using the NEBNext Ultra II Q5 master mix, the illumina universal primer, and specific octamer primers. The library was used for quantification with the QUBIT 3 Fluorometer using dS DNA HS reagent. The fragment analysis was performed on Agilent 2100 Bioanalyzer and loaded into Agilent DNA 7500 chip. This library was sequenced on the Illumina NextSeq platform and yielded a total of 6,071,188 reads and 2 × 150 bp paired-end read length. The raw illumina paired-end reads were filtered to remove low-quality reads and adapter region using Trim_Galore v0.6.4 and (Paired-End reAd mergeR) Pear (9, 10), with a minimum quality score of 30 and a minimum sequence length of 280. The quality of the reads was evaluated using FastQC v0.11.5 (11). The trimmed reads were used for *de novo* assembly using Unicycler v0.4.8 (https://github.com/rrwick/Unicycler) assembler (12). Contigs smaller than 500 bp, which typically represent sequencing artifacts, were removed, and the filtered assembly was used for the subsequent analysis. The genome assembly statistics were generated using QUAST (13). The completeness and contamination of the genome were assessed using CheckM v1.0.18 (14). Default parameters were used for all software tools employed in the analysis.

The strain S2 was identified as *Lactobacillus fermentum* using the Type strain Genome Server (15). Genome assembly of this strain revealed a genome size of 1,958,643, an N50 length of 60,574 bp, an L50 count of 12, a 919× genome coverage, and an average G + C content of 52%. Genome annotation was performed using the rapid prokaryotic genome annotation (PROKKA) (16) resulting in 83 contigs containing 1,949 CDS; 2,004 gene; 1 tmRNA; 3 rRNA genes; and 51 tRNA genes. *Lactobacillus fermentum* S2 genome is closely related to *Limosilactobacillus fermentum* ATCC 14931 with 94.1% dDDHd_4_. Rapid *in silico* analysis of the secondary metabolite biosynthesis gene clusters using antiSMASH v6.0.0 (17), PROKKA and PANZER 2 revealed the presence of genes involved in bile salt tolerance, acid tolerance, adhesion to epithelial cells, antioxidant production, and involved in the anti-inflammatory effects (thioredoxin reductase), heavy metal export channel, several types of uniporter symporter, antiporters, CRISPR array, biotin biosynthesis gene (biotin carboxyl carrier protein of acetyl-CoA carboxylase), amino acids biosynthesis gene, milk utilization gene (beta-galactosidase small and large subunit), and folate biosynthesis clusters (dihydrofolate synthase), suggesting the probiotic potential of strain S2.

## Data Availability

This whole-genome shotgun sequencing project has been deposited to the National Center for Biotechnology Information under the BioProject accession number PRJNA977258. The BioSample accession number is SAMN35523141. Raw sequencing data have been deposited in the NCBI Sequence Read Archive database under the accession number SRR24822339.
